# LSD1 Promotes Immune Exclusion and Suppresses IFN-γ-Associated Transcriptional Programs in Triple-Negative Breast Cancer

**DOI:** 10.3390/cancers18182920

**Published:** 2026-09-09

**Authors:** Dong Yeul Lee, Jing Han Hong, Peiyong Guan, Bernett Lee, Jianzhou Cui, Hui Xin Lau, Joe Poh Sheng Yeong, Puay Hoon Tan, Jinmiao Chen, Bin Tean Teh, Jabed Iqbal

**Affiliations:** 1 Department of Anatomical Pathology, Singapore General Hospital, Singapore 169856, Singaporejoe.yeong.p.s@sgh.com.sg (J.P.S.Y.); 2 Cancer and Stem Cell Biology Programme, Duke-NUS Graduate Medical School, Singapore 169857, Singapore; jinghan.hong@duke-nus.edu.sg (J.H.H.);; 3 National Cancer Centre Singapore, Singapore 168583, Singapore; p.g@u.duke.nus.edu; 4 Centre of Biomedical Informatics, Nanyang Technological University, Singapore 636921, Singapore; bernett.lee@ntu.edu.sg; 5 Immunology Translational Research Program, Yong Loo Lin School of Medicine, National University of Singapore, Singapore 117597, Singapore; jianzhou.cui@u.nus.edu (J.C.);; 6 Immunology Program, Life Sciences Institute, National University of Singapore, Singapore 117510, Singapore; 7 NUS-Cambridge Immunophenotyping Centre, National University of Singapore, Singapore 117510, Singapore; 8 Institute of Molecular and Cell Biology, Agency of Science, Technology and Research (A*STAR), Singapore 138634, Singapore; 9 Luma Medical Centre, Singapore 329565, Singapore; 10 Department of Microbiology and Immunology, Yong Loo Lin School of Medicine, National University of Singapore, Singapore 117597, Singapore; 11 Center for Computational Biology, Duke-NUS Graduate Medical School, Singapore 169857, Singapore; 12 Bioinformatics Institute, Agency of Science, Technology and Research (A*STAR), Singapore 138634, Singapore

**Keywords:** triple-negative breast cancer, LSD1/KDM1A, immune exclusion, interferon-signaling-associated signature, patient-derived organoids, epigenetic therapy, tumor microenvironment

## Abstract

**Background:** Triple-negative breast cancer (TNBC) exhibits aggressive biology and heterogeneous immune infiltration. Lysine-specific demethylase 1 (LSD1/KDM1A) is an epigenetic regulator implicated in oncogenic transcriptional programs, but its role in immune exclusion remains incompletely defined. **Methods:** LSD1 expression was evaluated by immunohistochemistry in TNBC specimens and correlated with survival. Functional effects of LSD1 depletion or pharmacological inhibition were assessed in TNBC cell lines and patient-derived xenograft organoids (PDXOs). Transcriptomic, ChIP-seq, multiplex immunofluorescence, and GeoMX digital spatial profiling analyses were integrated to characterize LSD1-associated transcriptional, chromatin, and immune programs. PBMC-PDXO co-cultures evaluated the effects of LSD1 inhibition on tumor growth and immune cell infiltration. **Results:** High LSD1 expression was associated with inferior overall survival, hypoxia-associated programs and suppressed immune pathways. LSD1-high tumors exhibited reduced infiltration of T cells, B cells, NKT-like cells, and macrophages. Spatial profiling revealed suppressed innate immune responses and type I interferon signaling within B-cell and T-cell compartments, respectively. ChIP-seq demonstrated preferential LSD1 occupancy at AP-1 motif-enriched regions, while co-immunoprecipitation supported association with AP-1 complexes. Integration of LSD1 chromatin occupancy sites with transcriptomics data identified *SPPL2A* and *NCOA3* as candidate LSD1-regulated genes associated with interferon-γ response pathways. LSD1 depletion increased SPPL2A, NCOA3, MHC-I and PD-L1 expression. LSD1 inhibition reduced PDXO growth and increased immune cell infiltration. **Conclusions:** LSD1 is associated with a poor clinical outcome and immune exclusion in TNBC. These findings support a role for LSD1 in AP-1-associated chromatin regulation and interferon-γ-related transcriptional response; they warrant further investigation into LSD1 inhibition as a therapeutic strategy for immune-cold TNBCs.

## Simple Summary

Triple-negative breast cancer is an aggressive form of breast cancer that often has limited treatment options and can develop with an immune-cold phenotype, which may reduce the efficacy of immunotherapies. We investigated lysine-specific demethylase 1 (LSD1), a protein involved in transcriptional regulation, and whether it contributes to this immune-cold environment. Higher levels of LSD1 were associated with poorer patient outcomes and fewer immune cells within the tumor microenvironment. LSD1 inhibition slowed tumor growth and increased immune cell infiltration in patient-derived tumors. These findings suggest that LSD1 may help cancer cells evade immune surveillance. Targeting LSD1 may therefore offer a potential therapeutic strategy for TNBCs with limited immune infiltration and provide a rationale for combining LSD1 inhibitors with immunotherapy.

## 1. Introduction

Triple-negative breast cancer (TNBC) accounts for 15–20% of all breast cancers and is characterized by aggressive clinical behavior, high rates of early recurrence, and limited targeted therapeutic options [1]. Although immune checkpoint blockade has improved outcomes in a subset of patients, many TNBC tumors exhibit an immune-excluded or “cold” phenotype with poor T-cell infiltration, resulting in limited immunotherapy responses [2,3]. Emerging evidence has also highlighted that dynamic epigenetic dysregulation, including aberrant chromatin conformation and altered gene regulatory element activity, plays critical roles in regulating both immune surveillance and tumor immune evasion [4,5].

Lysine-specific demethylase 1 (LSD1/KDM1A) is a histone demethylase that removes mono- and dimethyl groups from H3K4 and H3K9. Specifically, LSD1 can modulate transcriptional activation or repression depending on its associated protein complexes [6,7]. Additionally, LSD1 can function as a central regulator of transcription factor networks via the modulation of enhancer activity and interaction with lineage-specific transcription factors in other malignancies [8], where its broader role in orchestrating transcription factor cooperation and tumor–immune crosstalk in TNBC remains incompletely understood. Previous studies have established multiple oncogenic functions of LSD1 in TNBC, including promotion of tumor progression, metastasis, and therapeutic resistance [9,10]. In a genetically engineered mouse model, mammary-specific LSD1 knockout in a Brca1-deficient background significantly reduced tumor incidence, revealing a critical role of LSD1 in TNBC pathogenesis [11]. This effect was mediated, in part, through LSD1-mediated histone demethylation and silencing of the tumor-suppressor gene Tissue Factor Pathway Inhibitor 2 (*TFPI2*). Furthermore, LSD1 inhibition increased T-cell-attracting chemokines, enhanced CD8+ T-cell infiltration, and improved efficacy of chemotherapy and immune-checkpoint blockade [12]. Consistently, LSD1 expression is inversely correlated with cytotoxic T-cell chemokines (CCL5, CXCL9, CXCL10) and PD-L1 levels in TNBC specimens. While previous studies have reported LSD1 to function as a central regulator of transcription factor networks via modulation of enhancer activity and interaction with lineage-specific transcription factors in other malignancies [8], its broader role in orchestrating transcription factor cooperation and tumor–immune crosstalk in TNBC remains incompletely understood.

Despite these important observations, several aspects of LSD1 biology in human TNBC remain unresolved [13,14]. First, the clinical significance of LSD1 in shaping the immune landscape of TNBC has not been systematically examined in large patient cohorts. Second, whether LSD1 contributes to immune exclusion through coordinated regulation of chromatin-associated transcription programs remains unclear. Finally, although LSD1 has been shown to interact with lineage-specific transcriptional factors in other malignancies, the identity of its transcriptional partners and direct immune-associated transcriptional targets in TNBC remains largely undefined.

In the present study, we combined clinical profiling, multiplex immunofluorescence, digital spatial transcriptomics, chromatin immunoprecipitation sequencing, and functional patient-derived organoid models to define the role of LSD1 in TNBC. We demonstrated that elevated LSD1 expression is associated with poor clinical outcomes and widespread immune exclusion in TNBC. Mechanistically, we identified AP-1 enriched chromatin occupancy as a feature of LSD1 binding and nominate *SPPL2A* and *NCOA3* as direct transcriptional targets associated with interferon-responsive transcriptional programs. Finally, we present that LSD1 inhibition enhances tumor immunogenicity and suppresses tumor growth in clinically relevant TNBC models, supporting LSD1 as a potential therapeutic target for overcoming immune evasion in TNBC.

## 2. Materials and Methods

### 2.1. Local Cohort Study Design

A total of 389 archival formalin-fixed paraffin-embedded (FFPE) TNBC patient specimens diagnosed between 2003 and 2014 at the Department of Anatomical Pathology, Singapore General Hospital, were analyzed. Fifty-two (52) cases were excluded due to depleted tumor regions and/or IHC staining artefacts. All samples were obtained before patients underwent chemo-/radio-therapy; reception of neoadjuvant therapy was an exclusion criterion. Follow-up data were obtained from medical records. Disease-free survival (DFS) and overall survival (OS) were defined as the time from diagnosis to recurrence or death/date of last follow-up, respectively. Differentially expressed genes (DEGs) between sample groups were identified using Student’s *t*-tests with Welch’s correction (*p*-value < 0.05) and used for downstream ORA pathway analysis.

### 2.2. Public Cohort Data

All transcriptomics and survival data for breast cancer patients (with “PR”, “ER” and “HER” negative expression) were obtained from publicly available databases. The Cancer Genome Atlas (TCGA) information were obtained from cBioPortal (https://www.cbioportal.org/) [15,16]. Differentially expressed genes (DEGs) between sample groups were identified using Student’s *t*-tests with Welch’s correction (*p*-value < 0.01) and used for downstream ORA analysis.

### 2.3. Cell Lines

All commercial cell lines (MCF10A, MCF7, MDA-MB-231 and MDA-MB-468) were purchased from ATCC. MCF10A was maintained in DMEM/F-12 (Gibco, Grand Island, NY, USA) with 10% FBS, supplemented with 20 ng/mL EGF, 0.5 mg/mL hydrocortisone and 10 µg/mL insulin. MCF7, MDA-MB-231 and MDA-MB-468 were grown in DMEM (Gibco, Grand Island, NY, USA) with 10% FBS. All cells were cultured at 37 °C with 5% CO_2_.

### 2.4. Drug Compounds

LSD1 inhibitors, SP2509 (T2304, Targetmol, Wellesley Hills, MA, USA) and phenelzine (P6777, Sigma-Aldrich, St.Louis, MO, USA), were dissolved in DMSO. Unless indicated, cells were incubated with different concentrations for 72 h.

### 2.5. RNA Isolation and Quantitative PCR (qPCR)

Cell lines were treated as described and total RNA was extracted via a Direct-zol RNA Kit or TRIzol reagent as per the manufacturer’s protocols. RT was performed using iScript^TM^ Reverse Transcription Supermix for RT-qPCR as per the manufacturer’s protocols. Quantitative PCR was set up using BioRad SsoFast EvaGreen Supermix and the BioRad CFX96^TM^ Real-Time PCR System. qPCR was performed in biological triplicates and repeated at least thrice. Relative gene expression was analyzed using the 2^−ΔΔ^*^C^*^t^ method and normalized to *GAPDH*. The primer sequences used for qPCR were GAPDH: 5′–GTCTCCTCTGACTTCAACAGCG–3′ and 5′–ACCACCCTGTTGCTGTAGCCAA–3′; LSD1: 5′–TCAGGAGTTGGAAGCGAATCCC–3′ and 5′–GTTGAGAGAGGTGTGGCATTAGC–3′; NCOA3: 5′–GGACTAAGCAACAGGTGTTTCAAG–3′ and 5′–ACTGGAGGACTTGAGCCAACAG-3′; SPPL2A: 5′–TGCGTCTGGAAATGGCACAACC-3′ and 5′–GGTTGCATAGTGGTGTGGAAGTC–3′.

### 2.6. Patient-Derived Xenograft Organoid (PDXO)

Organoid models from PDX derived from breast tumors were established as described [17]. TNBC PDX organoid (BR5377B) was seeded in 3D hydrogel (Proprietary, Crown Bioscience Netherlands B.V., Leiden, The Netherlands) in 384-well plates (Greiner Bio-One B.V., Alphen aan den Rijn, The Netherlands) and incubated for approximately 72–96 h (at 37 °C with 5% CO_2_). Thereafter, either drug treatment alone or in combination with immune co-culture conditions was administered and maintained for an additional 96 h. Human peripheral blood mononuclear cells (PBMCs) were obtained from buffy coats from healthy volunteers retrieved from the Dutch blood bank (Sanquin, Amsterdam, The Netherlands), isolated by density-gradient centrifugation using Ficoll-Paque (GE Healthcare, Uppsala, Sweden), and cryopreserved until experiment execution. Thawed PBMCs were stained with cell tracker (Cat #C2925, CellTracker Green CMFDA Dye, Invitrogen/Thermo Fischer Scientific, Carlsbad, CA, USA) and added on top of the 3D hydrogel with a 5:1–10:1 E:T ratio. This E:T ratio was estimated based on organoid size and number vs. PBMC numbers. Co-cultures were treated with Staphylococcal enterotoxin A (SEA) (Cat #S9399, Sigma-Aldrich, St.Louis, MO, USA) as a positive control for the assay as it strongly activates human lymphocytes and promotes immune cell infiltration into tumoroids. At the endpoint, cultures were fixed with 4% formaldehyde (Sigma-Aldrich, St.Louis, MO, USA) and permeabilized with Triton-X100 (Sigma-Aldrich, St.Louis, MO, USA), prior to staining with Rhodamine-phalloidin (Sigma-Aldrich, St.Louis, MO, USA) and Hoechst 33258 (Sigma-Aldrich, St.Louis, MO, USA) overnight at 4 °C. Z-stack imaging was done using Molecular Devices ImageXpress Micro XLS (Molecular Devices, San Jose, CA, USA). Image analysis was performed using Ominer^®^ software (Crown Bioscience Netherlands B.V., Leiden, The Netherlands).

### 2.7. Tissue Microarray (TMA) Construction

Tumor regions for TMA construction were selected based on the pathological assessment of >50% of the sample being tumor area. For each sample, two or three representative tumor cores of 1 mm diameter were transferred from donor FFPE tissue blocks to recipient TMA blocks using an MTA-1 Manual Tissue Arrayer (Beecher Instruments, Sun Prairie, WI, USA). TMAs were constructed as previously described [18].

### 2.8. Immunohistochemistry (IHC) Analysis

TMA sections of 4 µm thickness were incubated with antibodies specific for LSD1 (clone 1B2E5, Thermo Fischer Scientific, Waltham, MA, USA). Details of the LSD1 antibody, labeling pattern, controls, and dilution factors are listed in Supplementary Table S1.

The scoring of antibody-labeled sections was carried out for nuclear LSD1 positivity. To generate the score, images of the labeled slides were captured using an IntelliSite Ultra-Fast Scanner (Phillips, Eindhoven, The Netherlands) before independent scoring by two trained pathologists blinded to the clinicopathological and survival information. Immuno-scoring was performed to determine the staining intensity and percentage of tumor cells stained in each TMA core. Where discordant, the cases were reviewed, and a consensus score was given. The H-score for LSD1 expression in each TMA was scored and calculated as follows: (3 × % of strong and complete nuclear staining in >30% of tumor cells) + (2 × % of moderate and complete nuclear staining in >10% of tumor cells) + (1 × % of faint/weak nuclear staining in <10% of tumor cells) [19]. An H-score of >2.5 (20% percentile cutoff) for LSD1 nuclear staining was categorized as positive, and tumors were stratified into “LSD1 positive” and “LSD1 negative” subsets.

### 2.9. RNA-Seq Pre-Processing

RNA was extracted from cell lines using Qiagen Rneasy Kits (Qiagen, Hilden, Germany). RNA quality was checked using Nanodrop (Thermo Fischer Scientific, Waltham, MA, USA) and Agilent 2100 Bioanalyzer (Agilent Technologies, Santa Clara, CA, USA). Libraries were made from good-quality RNA and sequenced to 40 million reads from Novaseq 600 at 150 base pairs in paired-end mode. Resultant fastq files were mapped to hg38, GENCODE annotation (v38) using nf-core/rnaseq (v3.8.1) [20,21], which maps the reads using STAR [22], and expression levels were quantified with RSEM [23]; biological duplicates were performed.

### 2.10. ChIP-Seq Data Analysis

ChIP-seq was performed as previously described [24]. Roughly 20 million cells were cross-linked with 1% formaldehyde for 10 min at room temperature and then quenched with glycine. Cells were then lysed and sonicated for 16 cycles (high setting—30 s on, 30 s off). The following antibody was used: LSD1 (# A300-215A, Bethyl Laboratories/Thermo Fischer Scientific, Montgomery, TX, USA). An amount of 20 μg of antibody was used for each ChIP. The fragmented chromatin with incubated with Protein-G Dynabeads (Life Technologies/Thermo Fischer Scientific, Carlsbad, CA, USA) that had been conjugated with antibodies for protein-of-interest and overnight at 4 °C. The beads with the bound protein cross-linked with chromatin were then washed before elution of the bound DNA. An amount of 10 ng of the amplified DNA was used to prepare libraries using the NEBNext ChIP-seq library preparation kit (New England Biolabs, Ipswich, MA, USA). Each library was sequenced to an average depth of 20 to 40 million raw reads on a Novaseq600 sequencer. The sequenced reads were checked for quality using FastQC. Trimmomatic was used to trim away adapter sequences. ChIP-seq data were pre-processed with nf-core/chipseq (v2.0.0) pipeline [20], which mapped reads to hg38 and performed peak calling using MACS2 [25] using an FDR cutoff of 0.01.

Confident peaks common between the two replicates of each cell line were retained for further analysis. A set of consensus peaks was defined by combining these confident peaks. DiffBind [26] was then used to identify the differential peaks by pairwise comparison of the three cell lines (FDR ≤ 0.01, |log_2_ fold-change| ≥ 1 and average normalized read counts (for either cell line or combined) ≥ 50). ChIPseeker [27] was used to annotate the genomic features of the LSD1 peaks. Motif enrichment was performed with findMotifsGenome.pl (-size 200 -mask -len 6,8,10,12 -p 5) in the HOMER software [28]. Pathway enrichment of the differential peaks was analyzed using rGREAT [29] on Hallmarks gene sets. LSD1 tracks were visualized with Integrative Genomics Viewer (IGV) [30].

### 2.11. GeoMX Digital Spatial Profiling (DSP)

TNBC specimens (TMA and full-slide sections) with LSD1 IHC status were used. Complete methods for GeoMX Digital Spatial Profiling in fixed assay were processed according to the GeoMX-DSP Manual Slide Preparation User Manual (MAN-10150, NanoString Technologies, Seattle, WA, USA) and GeoMX-DSP Spatial Proteogenomic Assay Protocol (MAN-101058, NanoString Technologies, Seattle, WA, USA), described in Merritt and colleagues [31]. Full-slide tissue sections of 5 µm thickness were stained with a 4-color fluorescence morphology marker panel targeting Syto13 (nuclear, 500 nM), CD3-AF532 (T-cell region; NBP2-54392AF532, c3e/1308, 1:100 dilution, Novus Biologicals, Centennial, CO, USA), CD8-AF594 (T-cell region; ab305370, CAL66, 1:100 dilution, Abcam, Cambridge, UK) and CD20-AF647 (B-cell region; NBP2-47840AF647, IGEL/773, 1:100 dilution, Novus Biologicals, Centennial, CO, USA). TMA tissue sections of 5 µm thickness were stained with a 4-color fluorescence morphology marker panel targeting Syto13 (nuclear, 500 nM), PanCK-AF488 (epithelial/tumor region; NBP2-33200AF488, AE1/AE3, 1:100 dilution, Novus Biologicals, Centennial, CO, USA), CD8-AF594 (T-cell region; ab305370, CAL66, 1:100 dilution, Abcam, Cambridge, UK) and CD3-AF647 (T-cell region; MCA1477A647, CD3-12, 1:100 dilution, Bio-Rad, Hercules, CA, USA). For Human Whole-Transcriptome Atlas RNA profiling, multicolored morphology markers enable compartment visualization for region-of-interest (ROI) selection by GeoMX software. Then, fluorescent antibody-conjugated UV-photocleavable oligonucleotides were exposed to UV light illumination for release and collected in 96-well plates. Library preparations were carried out and sequenced according to the GeoMX-DSP Spatial Proteogenomic Assay Protocol (MAN-101058, NanoString Technologies, Seattle, WA, USA). Raw counts were subjected to differential gene expression analysis using the “DESeq2” package [32], with fold change estimation values used for downstream GSEA analysis.

### 2.12. Plasmid Construction and Knockdown Line Generation

Human breast cell lines were transfected with pLKO.1 puro plasmid (Plasmid #8453, Addgene, Watertown, MA, USA), expressing shLSD1 (targeting 5′-GCTACATCTTACCTTAGTCATC–3′) or shNC (nontargeting control; Cat #SHC016, Sigma-Aldrich, St.Louis, MO, USA) using Lipofectamine^TM^ 3000 Transfection Reagent (Invitrogen/Thermo Fischer Scientific, Carlsbad, CA, USA), following the manufacturer’s protocol. After three days of transfection, protein lysates of cells were harvested for immunoblotting.

### 2.13. Protein Extraction and Immunoblotting

Cell lysates were prepared, and the protein concentration was measured using Bradford assay. Around 10 µg of total protein was used to run a 4–20% Mini-PROTEAN^®^ TGX^TM^ Precast Protein Gel (Bio-Rad, Hercules, CA, USA). A Trans-Blot Turbo System (Bio-Rad, Hercules, CA, USA) was used to transfer the protein onto the polyvinylidene difluoride (PVDF) membrane. The membranes were blocked in 5% nonfat milk and incubated overnight with primary antibodies specific for the following proteins: LSD1 (# A300-215A, 1:1000 dilution, Bethyl Laboratories/Thermo Fischer Scientific, Montgomery, TX, USA), GAPDH (10494-1-AP, 1:1000 dilution, Proteintech, Rosemont, IL, USA), HIF-1A (NB100-105, 1:1000 dilution, Novus Biologicals, Centennial, CO, USA), c-MYC (#9402, 1:1000 dilution, Cell Signaling Technology, Beverly, MA, USA), VEGFA (ab183100, 1:1000 dilution, Abcam, Cambridge, UK), Histone H3 monomethyl K4 (ab8895, 1:1000 dilution, Abcam, Cambridge, UK), Histone H3 dimethyl K4 (ab32356, 1:1000 dilution, Abcam, Cambridge, UK), Histone H3 trimethyl K4 (07-473, 1:1000 dilution, MilliporeSigma, Burlington, MA, USA), Histone H3 monomethyl K9 (ab8896, 1:1000 dilution, Abcam, Cambridge, UK), Histone H3 dimethyl K9 (ab1220, 1:1000 dilution, Abcam, Cambridge, UK), Histone H3 trimethyl K9 (ab8898, 1:1000 dilution, Abcam, Cambridge, UK), and Histone H3 (#4499, 1:1000 dilution, Cell Signaling Technology, Beverly, MA, USA). Primary antibodies were washed off and incubated with secondary antibodies (1:10,000 dilution, Promega, Madison, WI, USA) for one hour. Immunoreactions were detected with SuperSignal^TM^ West Femto Maximum Sensitivity Substrate (#34095, Thermo Fischer Scientific, Waltham, MA, USA) and imaged with a ChemiDoc Imaging System (Bio-Rad, Hercules, CA, USA).

### 2.14. Colony Formation Assays

An amount of 1 × 10^4^ cells per well were seeded in a 6-well plate and allowed to grow for 7–9 days. Colonies were stained with Crystal violet (Sigma-Aldrich, St.Louis, MO, USA) for visualization and scanned at 300 dpi using an HP scanner. Quantification of the total area of stained colonies was performed using ImageJ.

### 2.15. Flow Cytometry and Analysis

An amount of 3 × 10^5^ cells per well were seeded in a 6-well plate and allowed to adhere for 24 h. Cells were harvested and incubated with CD274-PE (#12-5983-42, 1:1000 dilution, eBioscience_^TM^_/Thermo Fischer Scientific, San Diego, CA, USA) or MHC Class I (#MA5-44137, 1:1000 dilution, Thermo Fischer Scientific, Waltham, MA, USA) antibodies for one hour, and analyzed using BD Arial II flow cytometry with the appropriate filters. Analysis was performed using FlowJo.

### 2.16. Pathway Enrichment Analysis

Over-representation analysis (ORA) or gene set enrichment analysis (GSEA) was conducted using clusterProfiler [33]. For ORA analysis, significant differentially expressed genes (DEGs) between the LSD1 status samples were analyzed through enrichGO and enrichKEGG functions. For gene ontology (GO) enrichment testing, all three GO categories (molecular function, biological process, cellular component) were selected, and a *p*-value < 0.05 was set as the cutoff criterion. For GSEA analysis, the fold change of gene expression between two groups was calculated, and the gene list was generated according to the log_2_FC values. Then, a GSEA-based enriched GO analysis was performed through gseGO function. All three GO categories (molecular function, biological process, cellular component) were selected, and a *p*-value < 0.05 was set as the cutoff criterion.

### 2.17. Statistical Analysis

Statistical analysis was performed using Rstudio running R version 4.2.3 (www.r-project.org) and GraphPad Prism 9.0 (GraphPad Software, Inc., San Diego, CA, USA). Survival outcomes were estimated with the Kaplan–Meier method and compared between groups using log-rank statistics. Statistical significance was defined by a *p*-value < 0.05.

## 3. Results

### 3.1. LSD1 Expression Correlates with Reduced Immune Infiltration and Inferior Survival in TNBC

**Figure 1:**
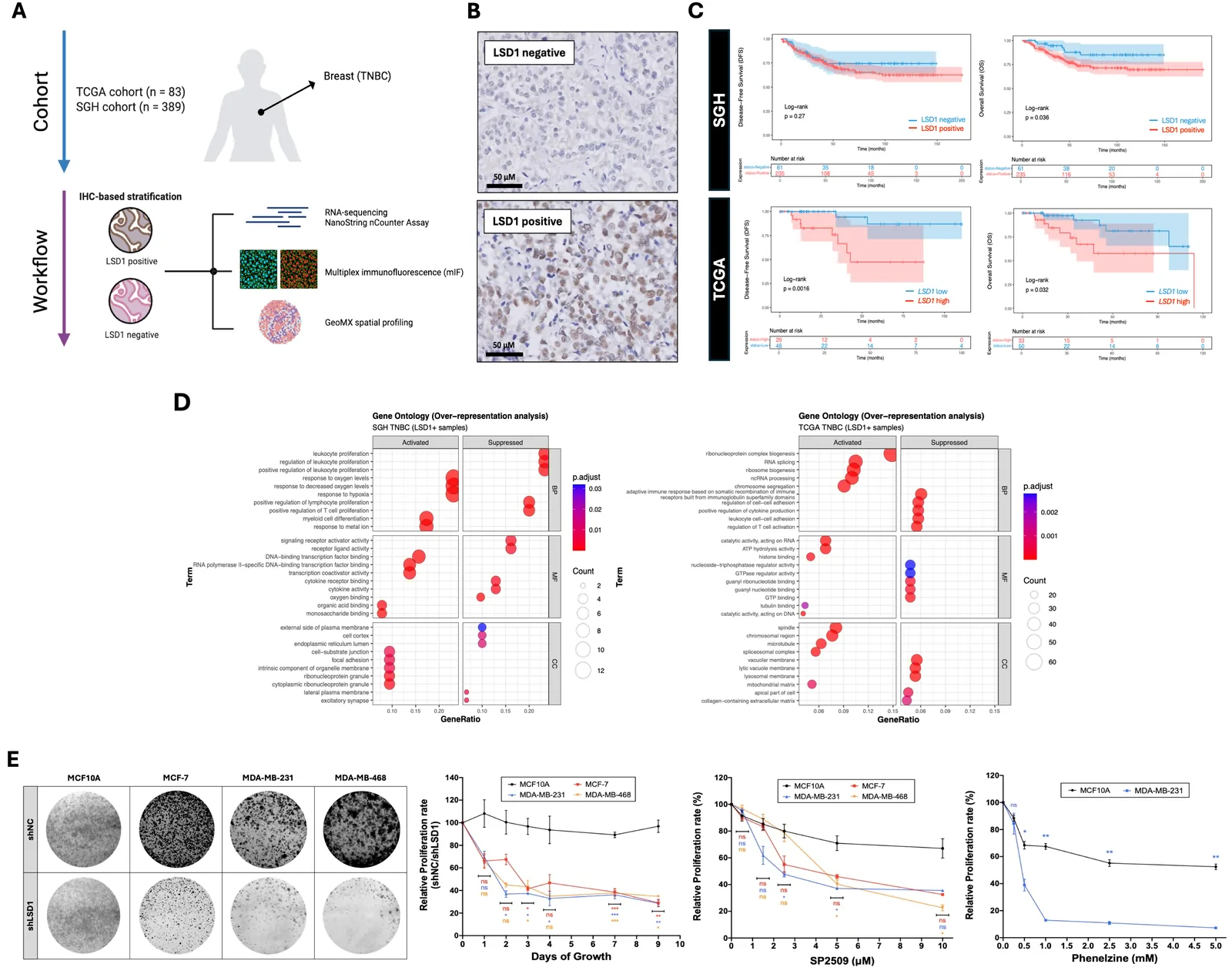
LSD1 expression is associated with poor prognosis, and LSD1 inhibition exerts anti-tumor effects in TNBC. (**A**) Flowchart of retrieval and stratification of LSD1-status in TNBC cohorts, subjected to molecular and functional analyses. (**B**) Representative LSD1 IHC staining in TNBC tissues. Scale bar, 50 μm. (**C**) Kaplan–Meier survival analysis indicates LSD1-positive patients to be significantly associated with poorer overall survival. (**D**) Top-10 activated/suppressed pathway analysis on differentially expressed genes in LSD1-positive patients from local SGH cohort (**left**) and public TCGA cohort (**right**). (**E**) LSD1 inhibition significantly reduces proliferation and colony formation capacity in breast cancer cells. Data for the curve are presented as mean ± SEM (*n* = 3); statistical significance was determined by 2-way ANOVA and multiple-comparison tests (Dunnett or Sidak); * *p* < 0.05, ** p < 0.01, *** *p* < 0.001.

To establish the clinical relevance and biological context of LSD1 in triple-negative breast cancer (TNBC), LSD1 immunohistochemistry was performed on a tissue microarray comprising 337 evaluable TNBC cases from Singapore General Hospital (2003–2014). Nuclear LSD1 expression was heterogeneous across tumors. Using a predefined H-score cutoff (>2.5, 20th percentile), LSD1 positivity was significantly associated with shorter overall survival compared to LSD1-negative cases (Figure 1A–C). Similarly, *LSD1*-high-stratified patients in the TCGA cohort exhibited significant poorer disease-free survival and overall survival (Figure 1C). To decipher the biological pathways associated with LSD1 expression, gene ontology analysis was performed on differentially expressed genes from LSD1-stratified patients (Figure 1D). Notably, LSD1-positive patients exhibit enrichment of hypoxic signatures and suppression of immune-related pathways, consistent with an immune-excluded tumor microenvironment associated with poorer clinical outcomes. To experimentally validate these clinical observations, LSD1 depletion in breast cancer cells reduced protein levels of hypoxia-associated factors (HIF-1α, VEGFA) and cell-cycle regulator MYC, while increasing global H3K4me1/2 and H3K9me1/2 levels, consistent with the demethylase activity of LSD1 (Supplementary Figure S1). Additionally, LSD1 inhibition resulted in significantly reduced colony formation and cell proliferation in breast cancer cells (Figure 1E). Notably, LSD1 inhibitors (SP2509 and phenelzine) significantly induced these inhibitory effects on MDA-MB-231 TNBC cells at concentrations above 2.5 µM and 500 µM, respectively. Together, these findings establish LSD1 as a poor prognostic marker associated with hypoxia and reduced immune activity in TNBC.

**Figure 2:**
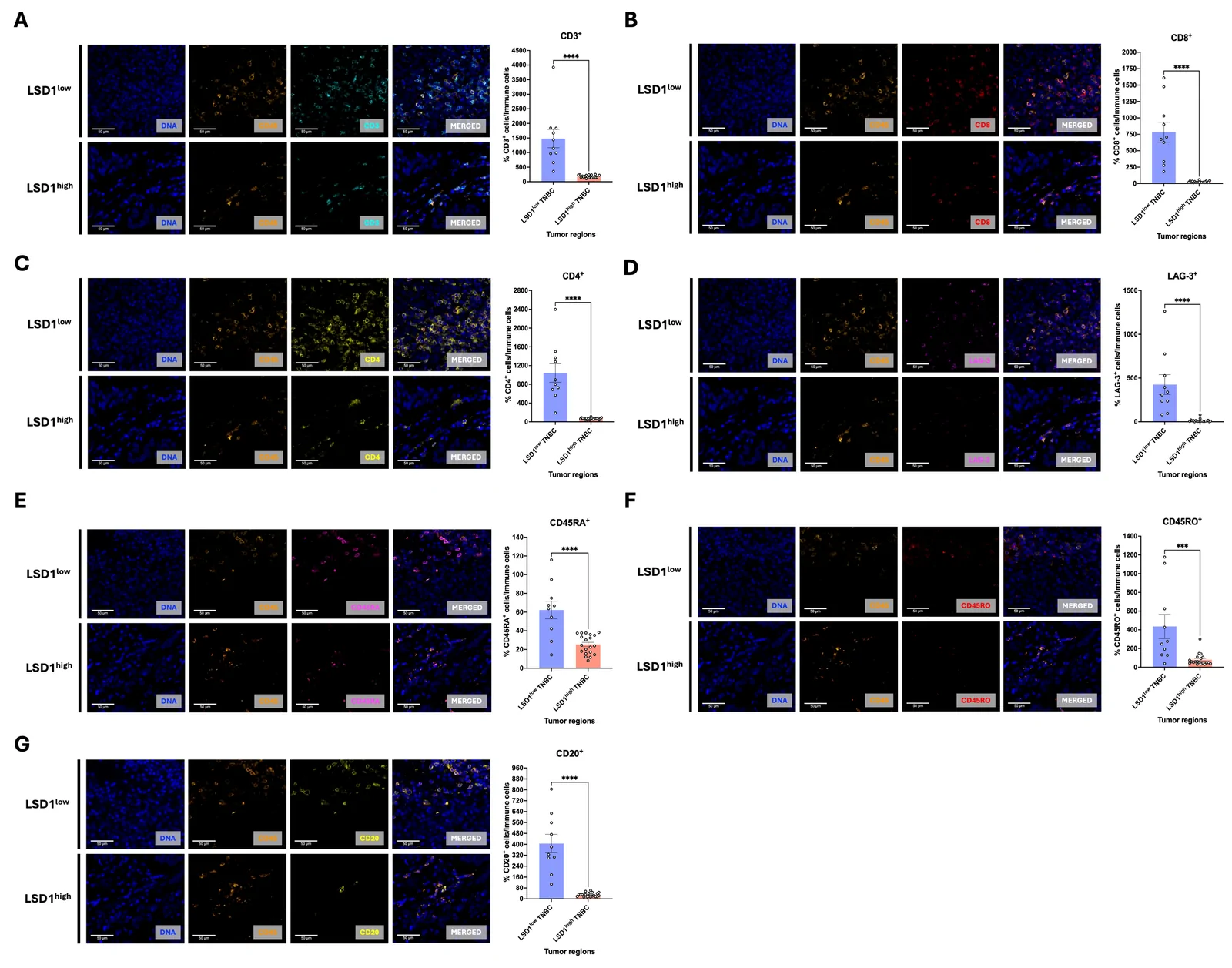

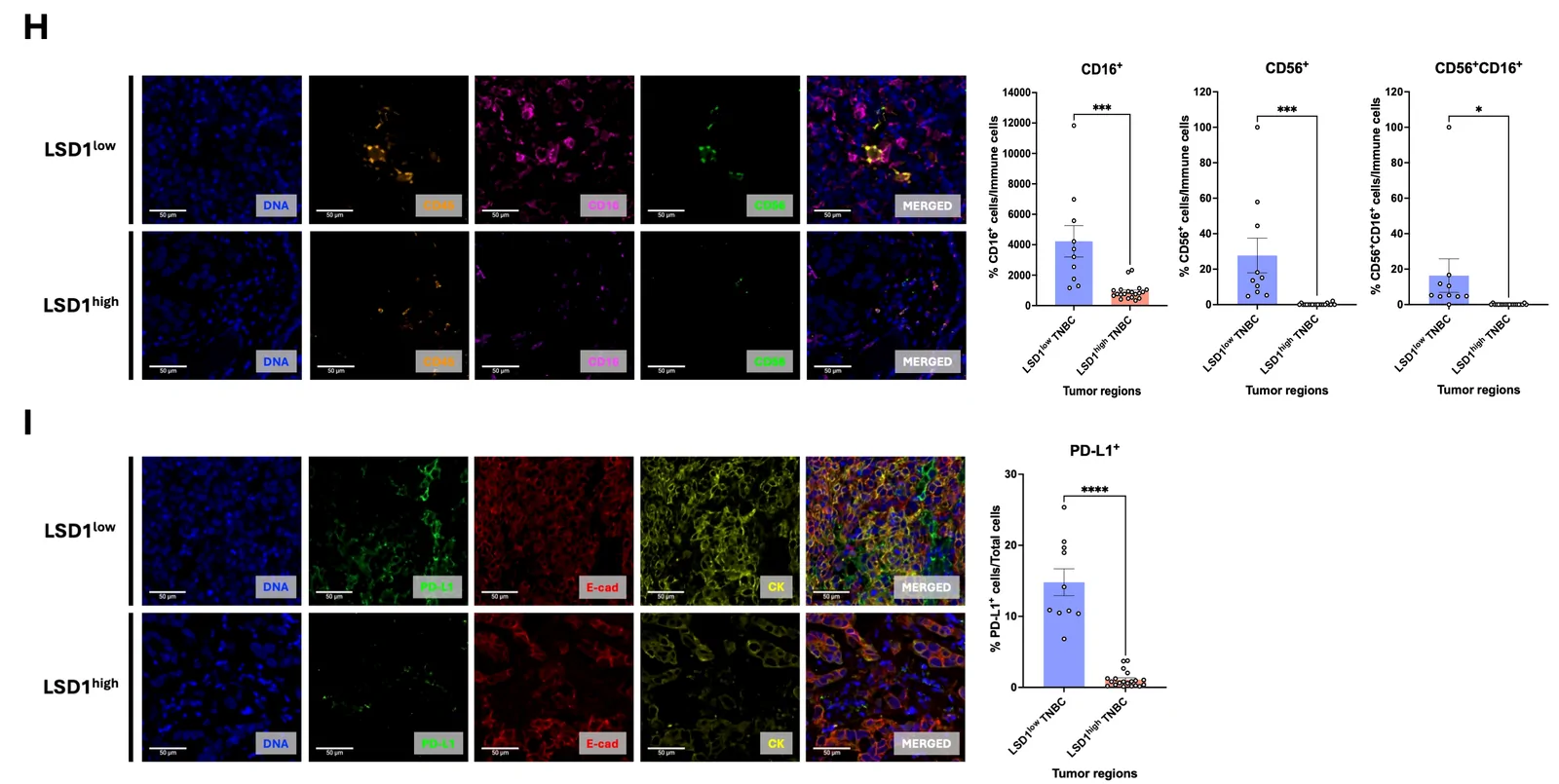
LSD1 positivity is associated with reduced intratumoral immune cell infiltration in TNBC. Quantification of percentage of intratumoral immune cell infiltration between LSD1-positive and LSD1-negative TNBCs (normalized to immune cells or total cells). (**A**) CD3+ T cells, (**B**) CD8+ T cells, (**C**) CD4+ T cells, (**D**) LAG3+ immune cell, (**E**) naïve T cell, (**F**) activated T cell, (**G**) CD20+ B cells, (**H**) CD56+CD16+ NKT cell, (**I**) intratumoral PD-L1 level. Data are presented as mean ± SEM (*n* > 3); statistical significance was determined using an unpaired two-tailed Student’s *t*-test; * *p* < 0.05, *** *p* < 0.001, **** *p* < 0.0001. Scale bar, 50 μm.

To characterize the tumor immune microenvironment, multiplex immunofluorescence was conducted on LSD1-stratified tumors. LSD1-high tumors exhibited reduced infiltration of CD3^+^, CD4^+^, and CD8^+^ T cells (including naïve and activated subsets), CD20^+^ B cells, CD3^+^CD56^+^ T/NKT-like cells, and CD68^+^ macrophages (Figure 2). Notably, PD-L1 expression was also lower in LSD1-high tumors, consistent with reduced baseline immune infiltration rather than an inflamed PD-L1-high phenotype. These observations indicate that high LSD1 expression is associated with widespread immune exclusion across multiple immune cell compartments.

**Figure 3:**
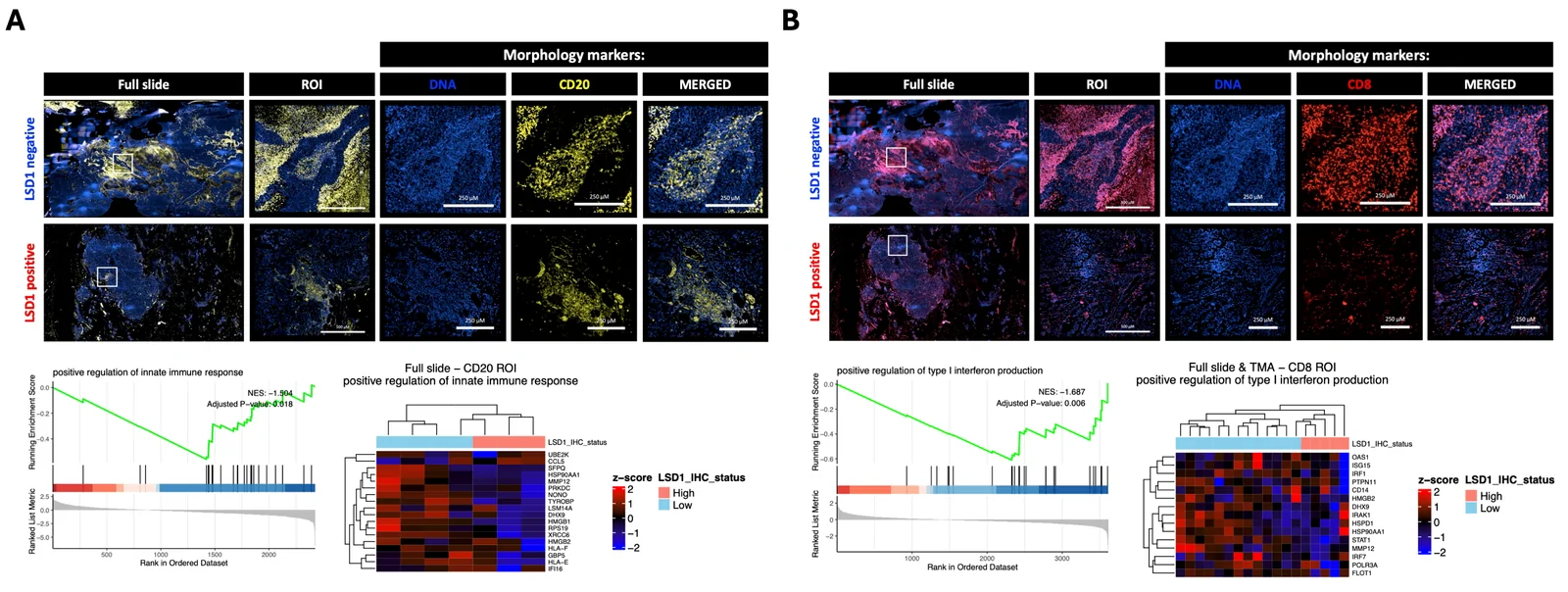
LSD1 positivity suppresses immunogenic pathways in specific immune cell compartments. (**A**) CD20+ B cells in LSD1-positive tumors exhibit downregulation of innate immune response genes. (**B**) CD8+ T cells in LSD1-positive tumors exhibit downregulation of type 1 interferon production genes. Scale bar, 500 μm and 250 μm.

Additionally, GeoMX digital spatial profiling of LSD1-stratified TNBC tissues further revealed compartment-specific transcriptional differences. In CD20^+^ B-cell regions, LSD1 positivity was associated with downregulation of innate immune response genes (Figure 3A). In CD8^+^ T-cell regions, LSD1 positivity was associated with suppression of type I interferon signaling pathways (Figure 3B). These spatial findings suggest that LSD1-high status correlates with alterations in discrete immune compartments.

### 3.2. LSD1 Occupancy at AP-1-Enriched Chromatin Regions in TNBC and Physical Association with AP-1 Complex

**Figure 4:**
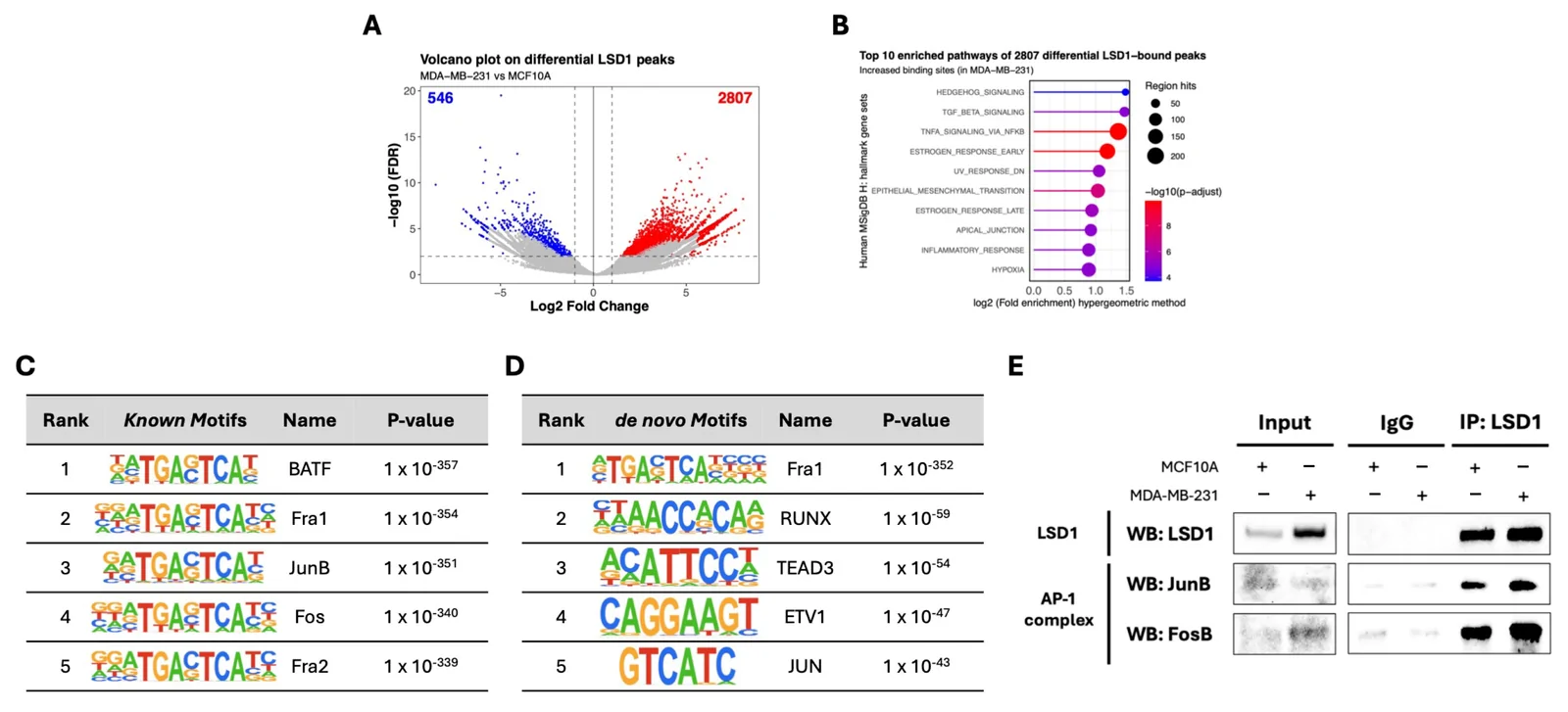
ChIP-seq analysis of LSD1 genome-wide binding in human breast cell lines. (**A**) Volcano plot on differential LSD1 peaks (between MDA-MB-231 and MCF10A). The x-axis represents log_2_ fold-change and the y-axis represents −log_10_(FDR). Genes meeting the criteria of FDR ≤ 0.01, |log_2_ fold-change| ≥ 1, and average normalized read counts ≥ 50 (for either cell line or the combined dataset) were considered significantly differentially expressed and highlighted accordingly. (**B**) Top-10 human MSigDB gene set enrichment analysis for 2807 differential LSD1-bound peaks. (**C**) Homer known motif analysis on 2807 enriched peaks. (**D**) Homer de novo analysis on 2807 enriched peaks. (**E**) Western blots showing co-immunoprecipitation of AP-1 complex proteins by an anti-LSD1 antibody in MDA-MB-231 and MCF10A. The uncropped blots are shown in Supplementary Materials.

Having established that LSD1-high TNBCs are associated with poor clinical outcome and reduced immune cell infiltration in the tumor microenvironment, we next investigated potential mechanisms underlying the observed clinical associations. We performed LSD1 ChIP-seq in TNBCs (MDA-MB-231) and comparator cell lines (MCF7 luminal and MCF10A normal-like mammary epithelial cells). A total of 2807 differentially increased genome-wide LSD1 binding sites were identified in TNBC cells (relative to LSD1 binding sites on MCF10A cells) (Figure 4A). Interestingly, genes associated with these LSD1 differential binding sites were significantly enriched for epithelial–mesenchymal transition (EMT), inflammatory response, and other tumor-associated pathways (Figure 4B).

To identify potential regulatory elements involved in LSD1-mediated gene regulation during disease progression, both known and de novo motif enrichment analyses were conducted for the 2807 bound sites, revealing significant enrichment of canonical AP-1 (JUN/FOS) binding motifs within LSD1-occupied regions in TNBC cells (*p* < 1 × 10^−6^) (Figure 4C,D). To determine whether LSD1 physically associates with AP-1 complex members, co-immunoprecipitation assays were performed (Figure 4E). Notably, immunoprecipitation of LSD1 resulted in robust co-precipitation of AP-1 proteins, supporting a physical association between LSD1 and the AP-1 complex. Collectively, these findings identify AP-1 as a candidate chromatin partner of LSD1 in TNBC.

### 3.3. Integration of ChIP-seq and Patient Transcriptomics Nominates Candidate Genes Associated with LSD1 Chromatin Occupancy and Differential Expression

**Figure 5:**
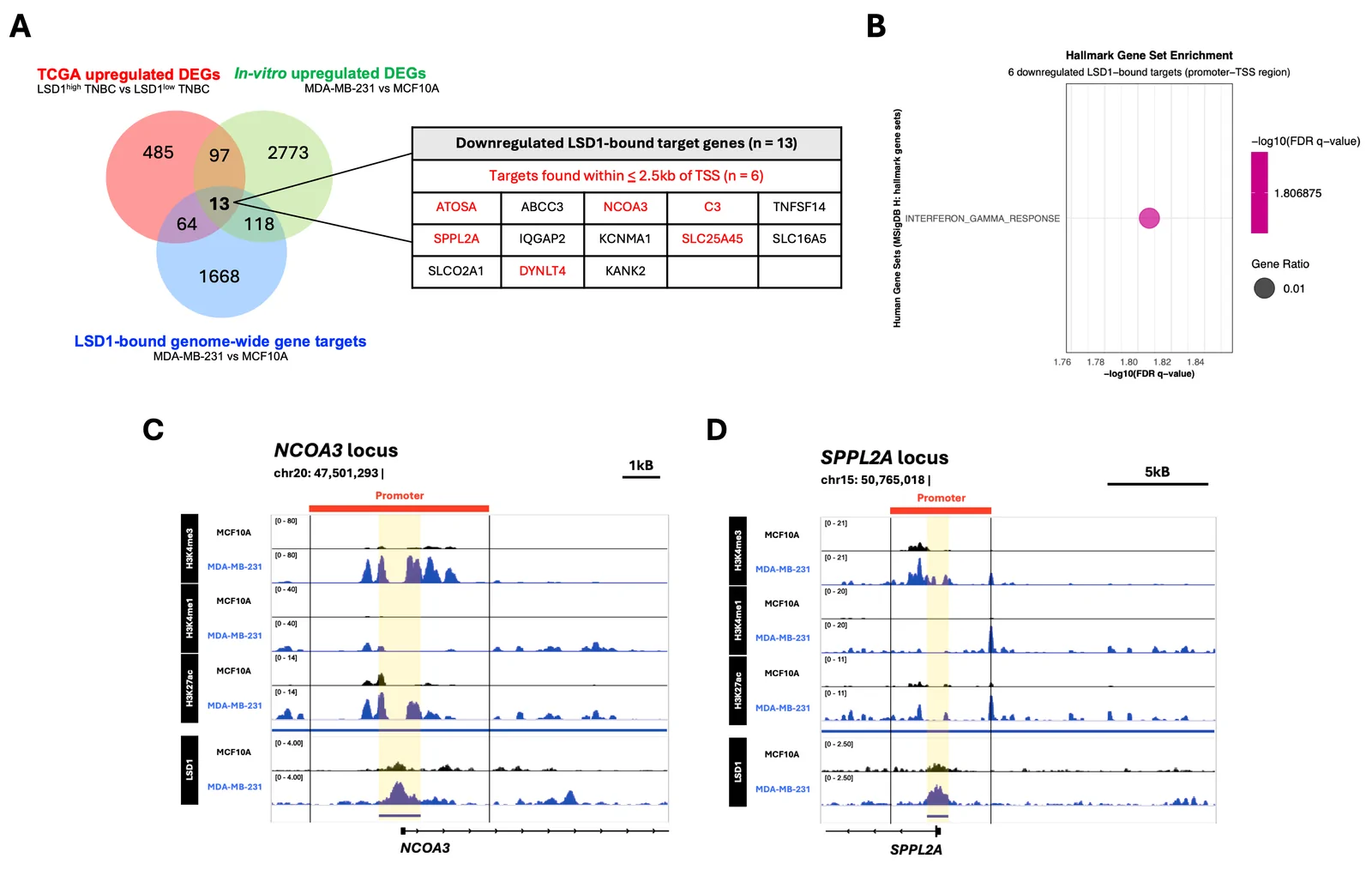
Identification of direct transcriptional targets of LSD1 involved in dampened immunogenicity. (**A**) Venn diagram of overlapping identified targets (from LSD1 ChIP-seq, in-vitro RNA-seq, LSD1-stratified patient RNA-seq). (**B**) Human MSigDB gene set enrichment analysis for six LSD1-bound gene targets at promoter-TSS regions. (**C**) LSD1 tracks for NCOA3 gene visualized by Integrative Genomics Viewer (lines indicate introns, boxes indicate exons, and arrowheads indicate direction of transcription). (**D**) LSD1 tracks for SPPL2A gene visualized by Integrative Genomics Viewer (lines indicate introns, boxes indicate exons, and arrowheads indicate direction of transcription).

Following the identification of LSD1-associated genome-wide bound regions, we next sought to explore potential links between LSD1 occupancy sites and their transcriptional regulatory programs involved in driving immune cell exclusion. To identify clinically relevant candidate targets, we intersected differential LSD1 ChIP-seq bound peaks (in TNBC cells), downregulated DEGs (in TNBC cells), and downregulated DEGs (in LSD1-high-stratified TCGA TNBC samples). This conservative approach yielded 13 putative downregulated LSD1-target genes (Figure 5A). Notably, six gene targets harbored differential LSD1 binding within the promoter region (defined as +2.5kb of TSS), suggesting potential direct transcriptional regulation by LSD1. Gene set enrichment analysis of these six promoter-associated target genes identified significant enrichment of the “interferon gamma response” pathway, with SPPL2A and NCOA3 among the leading candidate genes (Figure 5B). Consistent with these findings, LSD1 ChIP-seq occupancy tracks demonstrate prominent LSD1 enrichment at the promoter regions of SPPL2A and NCOA3 in MDA-MB-231 cells, further supporting these genes as potential direct transcriptional targets of LSD1 involved as mediators of altered immune-associated transcriptional programs during TNBC progression (Figure 5C,D). Overall, our integrative analyses identified SPPL2A and NCOA3 as direct transcriptional targets linking LSD1 chromatin occupancy to IFN-γ-associated transcriptional programs.

### 3.4. Validation of IFN-γ Candidate Targets and Effects on Immune Recognition Markers

**Figure 6:**
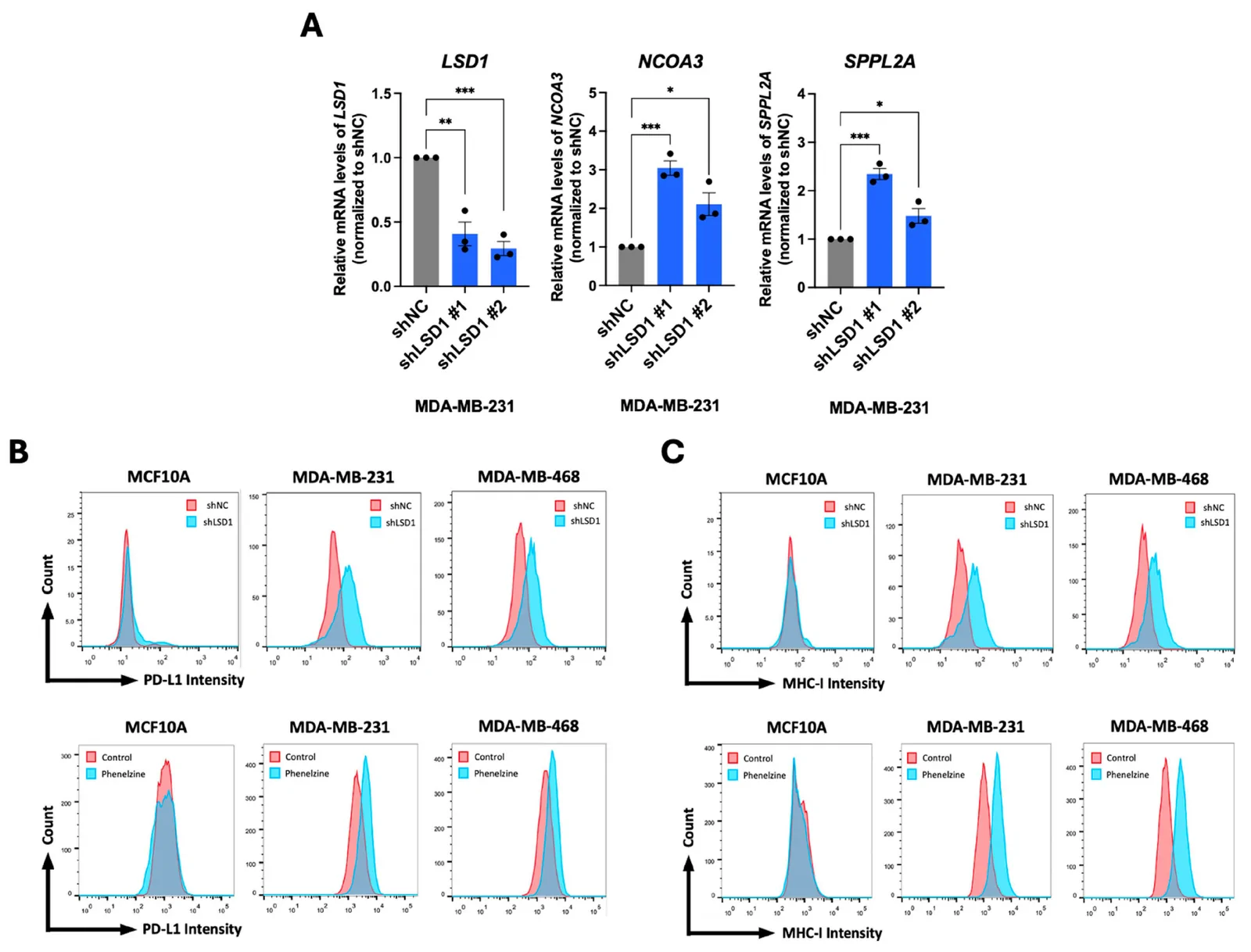
Validation of LSD1-regulated IFN-γ response-associated genes and immunogenic phenotype in TNBC. (**A**) Quantitative PCR (qPCR) analysis of *SPLL2A* and *NCOA3* mRNA expression following LSD1 knockdown in TNBC cells. Data are presented as mean ± SEM (*n* > 3); statistical significance was determined using an unpaired two-tailed Student’s *t*-test; * *p* < 0.05, ** p < 0.01, *** *p* < 0.001. (**B**) Flow cytometry quantification of cell-surface PD-L1 expression following LSD1 knockdown in TNBC cells. (**C**) Flow cytometry quantification of cell-surface MHC-I expression following LSD1 knockdown in TNBC cells.

To validate the transcriptional regulation of these IFN-γ response-associated candidate genes by LSD1, we assessed the expression of SPPL2A and NCOA3 following LSD1 depletion in TNBC cells. Quantitative PCR (qPCR) analysis demonstrated that shRNA-mediated knockdown of LSD1 in TNBC cells significantly upregulated NCOA3 and SPPL2A (Figure 6A). These findings are consistent with the loss of LSD1-mediated transcriptional repression and further support SPPL2A and NCOA3 as direct transcriptional targets of LSD1. Restoration of SPPL2A and NCOA3 expression following LSD1 depletion is consistent with the enrichment of the IFN-γ response gene set identified in our integrative analyses and supports de-repression of this immune-associated transcriptional program.

To determine whetherthese transcriptional changes are translated into an altered immunogenic phenotype, we next examined the surface antigen presentation level and immune checkpoint molecules following LSD1 inhibition. Flow cytometric analysis demonstrated that both shRNA-mediated and pharmacological inhibition of LSD1 significantly increased cell surface levels of major histocompatibility complex class I (MHC-I) and programmed death-ligand I (PD-L1) in TNBC cells (Figure 6B,C). Together, these results suggest that LSD1 inhibition promotes an IFN-γ-responsive tumor-cell phenotype characterized by increased antigen presentation together with an adaptative induction of PD-L1 immune checkpoint expression.

### 3.5. LSD1 Inhibition Reduces TNBC Organoid Growth and Enhances Immune Cell Infiltration

**Figure 7:**
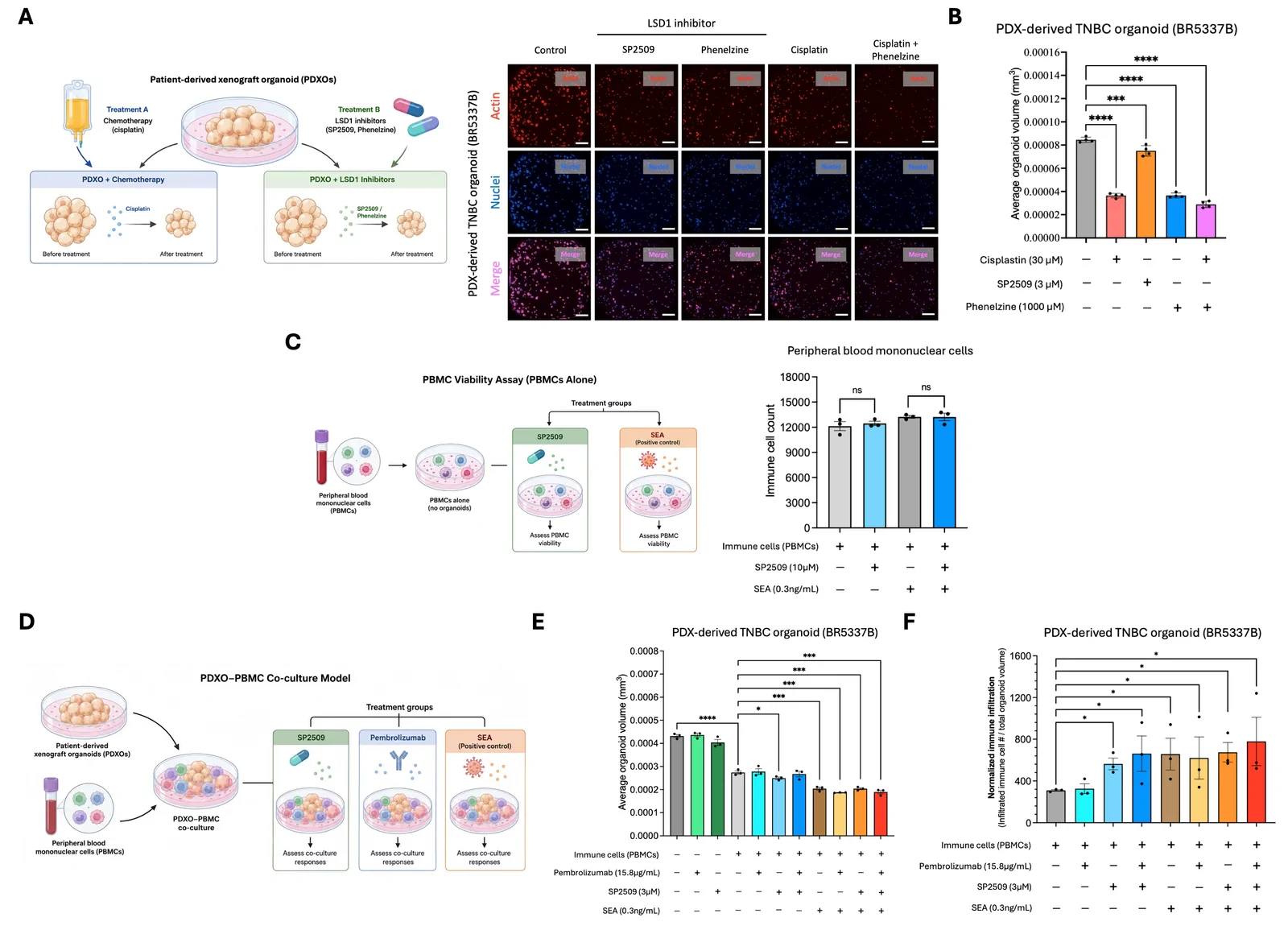
LSD1 inhibition effects on TNBC patient-derived xenograft organoids (PDXOs). (**A**) Graphical representation of LSD1 inhibitor (SP2509, phenelzine) treatment in monoculture TNBC PXDO model (BR5337B). Scale bar, 500μm. (**B**) LSD1 inhibitor (SP2509, phenelzine) alone or in combination with cisplatin significantly reduces tumor organoid volume in the TNBC PDXO model. Data are presented as mean ± SD (*n* = 4); statistical significance was determined by 1-way ANOVA and multiple-comparison tests (Dunnett or Sidak); *** *p* < 0.001, **** *p <* 0.0001. (**C**) Graphical representation of SP2509 treatment effect on PBMC viability. LSD1 inhibitor (SP2509) at maximum dose (10 µM), with or without SEA, exhibits no significant PBMC depletion. Data are presented as mean ± SEM (*n* = 3); statistical significance was determined by an unpaired two-tailed Student’s *t*-test. (**D**) Graphical representation of LSD1 inhibitor (SP2509), anti-PD-1 (Pembrolizumab), *Staphylococcal Enterotoxin A* (SEA) treatment in a tumor-immune co-culture model (BR5337B). (**E**) LSD1 inhibitor (SP2509) alone or in combination with pembrolizumab and/or SEA significantly reduces tumor organoid volume in a TNBC co-culture PDXO model. Data are presented as mean ± SEM (*n* = 3); statistical significance was determined by an unpaired one-tailed Student’s *t*-test; * *p* < 0.05, *** *p* < 0.001, **** *p <* 0.0001. (**F**) LSD1 inhibitor (SP2509) alone or in combination with pembrolizumab and/or SEA significantly induces immune infiltration in a TNBC co-culture PDXO model. Data are presented as mean ± SEM (*n* = 3); statistical significance was determined by a Mann–Whitney test; * *p* < 0.05.

Given the observed effects of LSD1 perturbation on immune-associated markers and tumor cell proliferation in vitro, we next sought to evaluate these findings in a more physiologically relevant model. We employed patient-derived xenograft organoids (PDXOs) in both monoculture and immune co-culture systems. In PDXO monoculture, LSD1 inhibitors (SP2509 and phenelzine), alone or in combination with chemotherapy, significantly reduced average organoid volume in the TNBC model (BR5337B) (Figure 7A,B). The anti-tumor effects of LSD1 inhibition were comparable to those observed with cisplatin, highlighting the therapeutic potential of LSD1-targeted treatments in TNBC.

To further investigate the potential immunomodulatory effects of LSD1 inhibition in an immune microenvironment, we next established a PBMC-PDXO co-culture model. Prior to co-culture experiments, we assessed the effects of SP2509 on PBMC viability and found that PBMC viability was maintained at the highest concentration (10 µM), supporting the use of SP2509 inhibitor in subsequent immune co-culture experiments (Figure 7C).

In PBMC-organoid co-culture assays, we examined the effects of SP2509 and pembrolizumab (anti-PD-1), alone or in combination, with SEA included as an immune-stimulatory control (Figure 7D,E). In the absence of immune cells, untreated PDXOs exhibited significantly larger average tumor volumes compared to PDXOs co-cultured with PBMCs, indicating that the presence of PBMCs exert an anti-tumor effect. Interestingly, SP2509 treatment significantly reduced tumor volume compared to PBMC co-culture alone, while pembrolizumab did not result in a significant reduction. SEA treatment resulted in a significant reduction in tumor volume compared with PBMC co-culture alone, consistent with its use as an immune-stimulatory control. Similar reductions in organoid volume were observed in the SEA-containing treatment groups, including SEA combined with pembrolizumab, SP2509, or both agents (in comparison with PBMC co-culture alone). These findings demonstrate that SP2509 retains anti-tumor activity in the presence of PBMCs and that immune stimulation further suppresses PDXO growth.

We next examined whether these treatment effects were associated with changes in immune cell infiltration into the PDXOs (Figure 7F). Compared with PBMC co-culture alone, immune cell infiltration was significantly increased following treatment with SP2509, pembrolizumab, or their combination. SEA-containing conditions (including SEA alone, SEA + pembrolizumab, SEA + SP2509, and SEA + SP2509 + pembrolizumab) similarly exhibited significantly increased immune cell infiltration compared with PBMC co-culture alone. Notably, the combination of SEA, SP2509, and pembrolizumab showed the highest level of normalized immune cell infiltration among the treatment groups.

Together, these findings indicate that LSD1 inhibition not only suppresses TNBC growth but is also associated with enhanced immune cell infiltration in a tumor-immune co-culture model. The increased immune cell infiltration observation following SP2509 treatment, together with its anti-tumor effects in a PBMC-PDXO co-culture, supports a potential role for LSD1 inhibition in modulating tumor-immune microenvironments and provides a rationale for LSD1 drugs in combination with immune checkpoint blockade. Overall, these results support the translational potential of LSD1 inhibition in clinically relevant TNBC models.

## 4. Discussion

This study provides an integrated clinical, spatial, molecular and functional analysis of LSD1 in triple-negative breast cancers and identifies an association between LSD1 expression and an immune-excluded tumor state. Across clinical specimens, high nuclear LSD1 expression was associated with inferior overall survival, reduced infiltration of multiple immune cell populations, and transcriptional programs characterized by hypoxia and suppression of immune-related pathways. These clinical observations align with and extend previous studies linking LSD1 in the regulation of anti-tumor immunity in breast cancer [11,12,34]. The association with hypoxia-related transcriptional programs is also notable, given the established relationship between hypoxia, tumor progression, and impaired anti-tumor immunity [35,36]. However, given that these clinical analyses are observational, whether LSD1 directly drives immune exclusion or instead marks an immune-cold tumor state remains to be established.

Thus, our chromatin and transcriptional analyses aimed provide a potential mechanistic context for these clinical associations. ChIP-seq profiling identified substantial increased LSD1 occupancy in TNBC cells, with significant enrichment of AP-1 binding motifs within differential LSD1-bound regions. Co-immunoprecipitation further demonstrated an association between LSD1 and AP-1 complexes, supporting the possibility that AP-1-associated chromatin regions represent an important context for LSD1 activity in TNBC. Consistent with this finding, recent work has demonstrated that LSD1 occupies AP-1 motif-enriched regulatory regions and regulates key immune-associated transcriptional programs in epithelial cells [37], suggesting that LSD1-AP-1 interactions may represent a broader mechanism of epithelial immune regulation. Additionally, integration of LSD1 occupancy sites (at promoter regions) with differential gene expression in TNBC cells and LSD1-stratified patient tumors identified SPPL2A and NCOA3 as candidate LSD1-regulated genes associated with interferon-γ response pathways. Previous studies have implicated SPPL2A in B-cell maturation and IFN-γ immune responses, where it is strongly involved in the activation of adaptive immunity [38,39,40,41]. Similarly, the inhibition of SRC-3 (*NCOA3*) generates anti-tumor immunity in breast tumors [42] given its key role in human Treg-mediated immune suppression [43,44,45,46]. Consistent with a repressive role for LSD1, LSD1 depletion increased SPPL2A and NCOA3 expression in TNBC cells. Together, these findings suggest SPPL2A and NCOA3 as potential direct transcriptional targets through which LSD1 may influence immune-associated transcriptional programs in TNBCs.

Functionally, genetic and pharmacological inhibition of LSD1 increased surface expression of MHC class I and PD-L1 levels in TNBC cells, reduced tumor growth in patient-derived organoids, and was associated with increased immune cell infiltration in PBMC-organoid co-cultures. Importantly, SP2509 treatment at maximal dose (10 µM) preserved total PBMC counts and immune cell viability, supporting the compatibility of LSD1 drugs with immune cells under the conditions examined. Previous studies have also reported that LSD1 loss can enhance chemokine expression, promote T-cell infiltration and improve responses to chemotherapy or immune checkpoint blockade in TNBC models [11,12,47]. Our findings extend these observations by linking LSD1 expression with immune-exclusion-associated phenotypes in clinical cohorts and demonstrating anti-tumor effects accompanied by increased immune cell infiltration in patient-derived models. Collectively, this multi-layered evidence is consistent with the possibility that LSD1-associated transcriptional and chromatin states contribute to an immune-cold phenotype in TNBC, characterized by reduced immune-associated and interferon-responsive programs. Conversely, LSD1 inhibition promoted an immune-associated tumor-cell phenotype, including increased MHC class I and PD-L1 expression, while retaining anti-tumor activity in an immune-organoid model. These findings provide a rationale for further development and investigation of LSD1-targeted therapies, including their potential combination with existing immune-based therapies, to address the unmet need in immune-cold TNBC.

## 5. Limitations

This study also has several limitations. First, the associations between LSD1 expression, hypoxia-related signatures, immune infiltration and survival are correlative; direct causality was not established due to the absence of in vivo functional experiments. Second, most mechanistic validation was performed in a single TNBC cell line (MDA-MB-231), and patient-derived organoid models were used for functional validation, additional cell lines will be required to establish the generalizability of the results. Third, phenelzine has off-target activities beyond LSD1 inhibition; therefore, emphasis was placed on results replicated with the more selective inhibitor SP2509. Fourth, the organoid–PBMC co-culture model provides an experimentally tractable immune-competent system but does not recapitulate the full complexity of the tumor microenvironment, including stromal compartments, tissue-resident immune populations, and the dynamic interactions among multiple immune cell subsets. The assays were short-term (96 h) and lacked autologous T-cell specificity or stromal components, limiting conclusions about long-term immune modulation. Finally, although LSD1 occupancy was enriched at AP-1 motifs and physical interaction with AP-1 complexes was demonstrated, the functional requirement of AP-1 for LSD1-dependent transcriptional regulation was not directly established. Similarly, additional direct gain- and loss-of-function studies on SPPL2A and NCOA3 candidate targets are required to determine their contribution to tumor immunity. Future studies incorporating additional patient-derived models, defined immune cell populations, mechanistic perturbation of candidate targets, and longer-term in vivo systems will be crucial to determine whether LSD1 inhibition can promote durable immune-mediated tumor control.

Despite these limitations, the multi-omics integration of clinical immunohistochemistry, spatial transcriptomics, chromatin profiling, and patient-derived 3D models provides a comprehensive characterization of LSD1-associated features in TNBC. These findings support LSD1 as a biomarker of aggressive, immune-cold disease and a candidate for further therapeutic exploration, particularly in combination strategies.

## 6. Conclusions

In summary, this study identifies LSD1 as a potential link between epigenetic regulation, tumor-intrinsic transcriptional programs, and immune exclusion in TNBC. High LSD1 expression was associated with poorer clinical outcomes, reduced immune infiltration, and hypoxia-related transcriptional programs, while chromatin profiling identified AP-1 motif enrichment at LSD1-bound regions. Functional studies further demonstrated that LSD1 inhibition altered tumor-cell immune-associated phenotypes, reduced growth of patient-derived organoids, and increased immune cell infiltration in an immune–organoid 3D model. Together, these findings provide a translational framework for further defining the mechanisms by which LSD1 contributes to immune-cold TNBC and support continued investigation of LSD1-targeted approaches, particularly in combination with immune-based therapies.

## Data Availability

RNA-seq, ChIP-seq data sets and local SGH human cohort data are available upon request from the corresponding author.

## Supplementary Materials

The following supporting information can be downloaded at: https://www.mdpi.com/article/10.3390/cancers18182920/s1, Figure S1: Immunoblot analysis of HIF-1, VEGFA, MYC and histone marks expression in human breast lines following lentiviral-mediated expression of shRNA targeting LSD1 or scramble (as negative control); Table S1: Details of antibodies used for immunohistochemistry labelling of TNBC tissues.
