# Supplementary material for: LSD1 Promotes Immune Exclusion and Suppresses IFN-γ-Associated Transcriptional Programs in Triple-Negative Breast Cancer
Source: Cancers (Basel). 2026 Sep 9;18(18):2920. doi: 10.3390/cancers18182920 (PMC13604181; doi:10.3390/cancers18182920)

Immunoblot for shLSD1 lines (part 1)

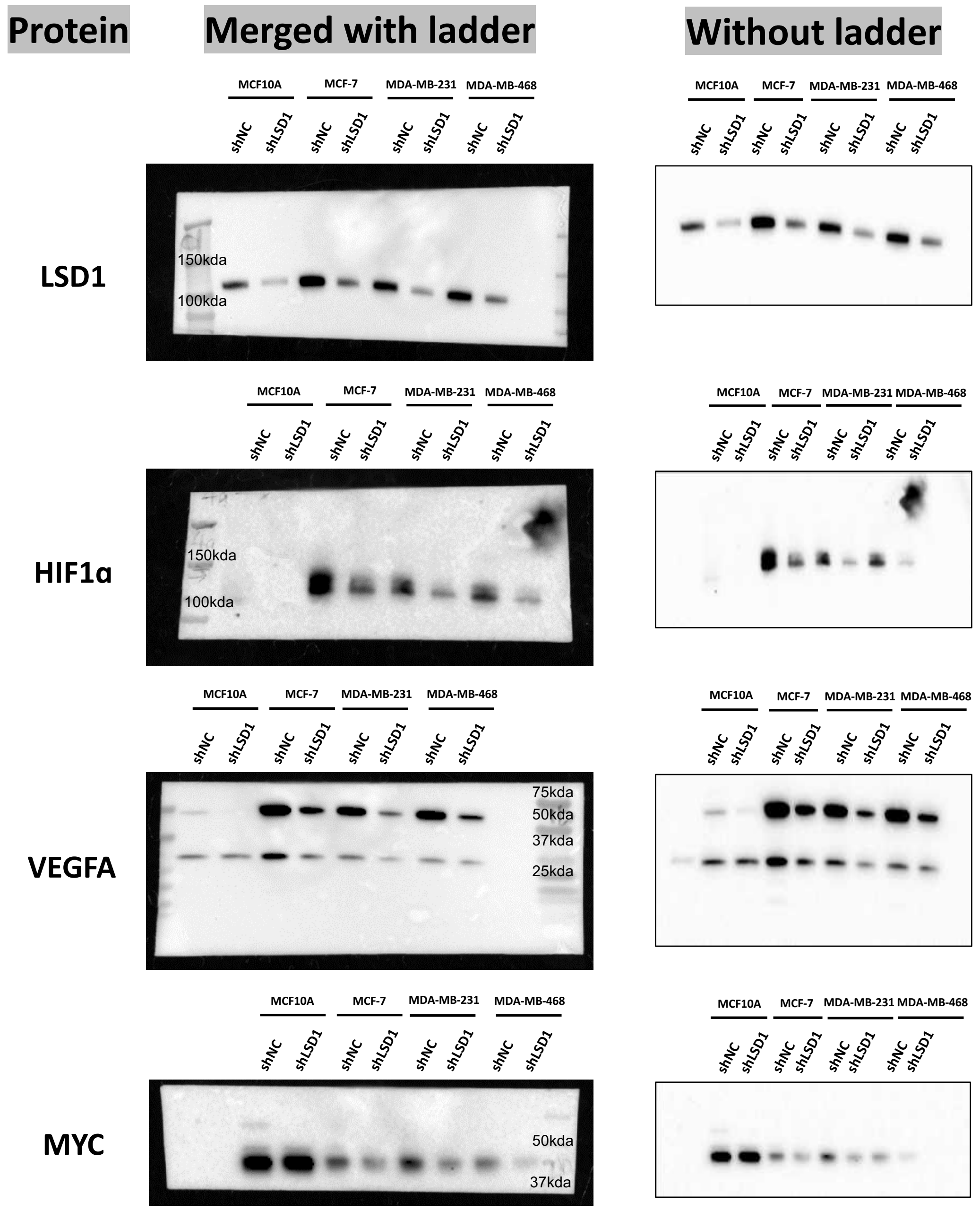

Immunoblot for shLSD1 lines (part 2)

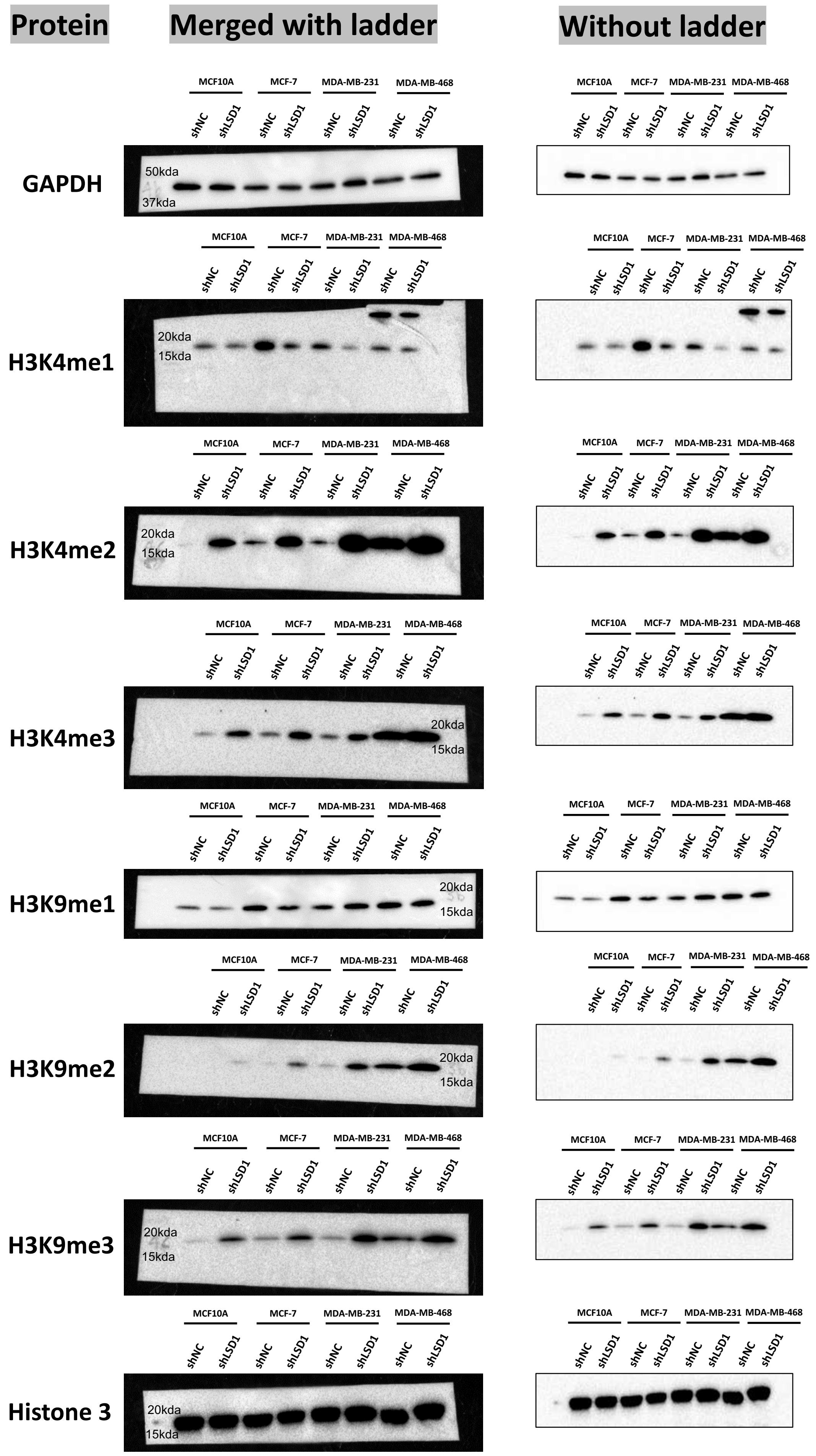

Immunoblot for input

Protein	Merged with ladder	Without ladder (only MCF10A/MDA-231)
**LSD1** (only MCF10A, MDA-MB-231 lanes were used)	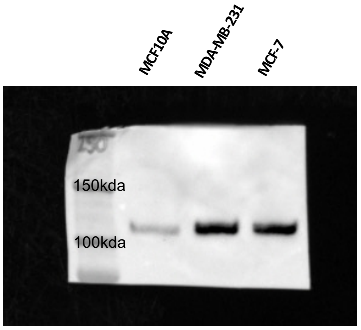	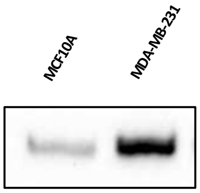
**JunB** (only MCF10A, MDA-MB-231 lanes were used)	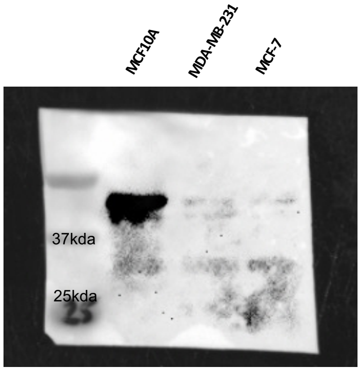	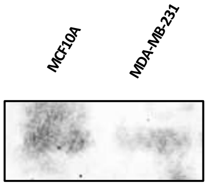
**FosB**	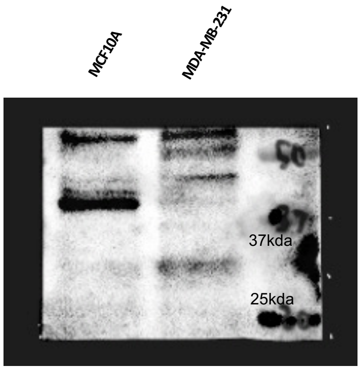	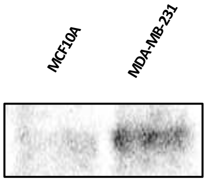

Immunoblot for co-IP

Protein | **Merged with ladder** | **Without ladder** (only MCF10A/MDA-231)

LSD1

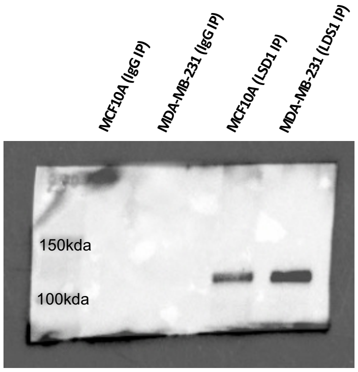

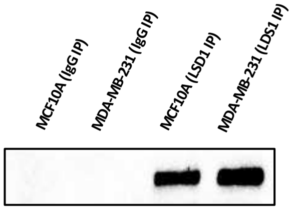

JunB
(only MCF10A, MDA-MB-231 lanes were used)

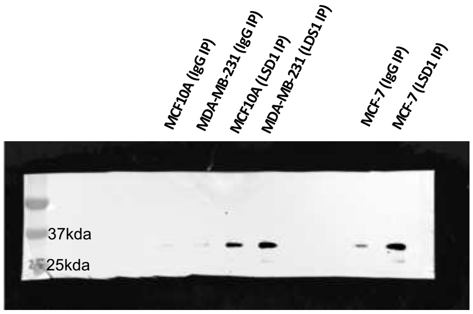

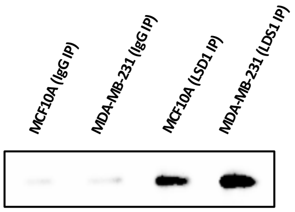

FosB
(only MCF10A, MDA-MB-231 lanes were used)

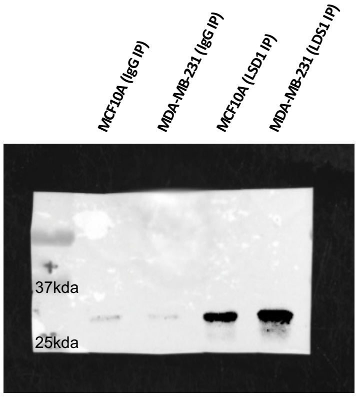

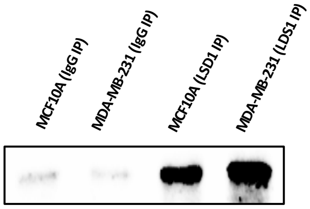

Supplement: Supplementary file 1 [file cancers-18-02920-s001.zip › cancers-4402649-original-images.pdf]
