# Supplementary material for: LSD1 Promotes Immune Exclusion and Suppresses IFN-γ-Associated Transcriptional Programs in Triple-Negative Breast Cancer
Source: Cancers (Basel). 2026 Sep 9;18(18):2920. doi: 10.3390/cancers18182920 (PMC13604181; doi:10.3390/cancers18182920)
Supplement: Supplementary file 1 [file cancers-18-02920-s001.zip › cancers-4402649-supplementary update.pdf]

Supplementary Figures and Table

Figure S1. Immunoblot analysis of HIF-1, VEGFA, MYC and histone marks expression in human breast lines following lentiviral-mediated expression of shRNA targeting LSD1 or scramble (as negative control).

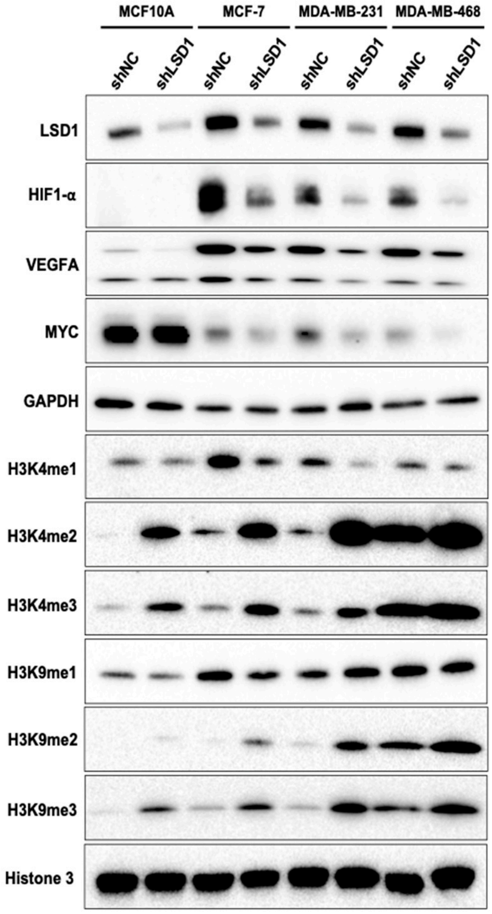

Table S1. Details of antibodies used for immunohistochemistry labelling of TNBC tissues

**Table S1.** Details of antibodies used for immunohistochemistry labelling of TNBC tissues						
**Antibody**	**Clone**	**Dilution**	**Source**	**Cellular localisation**	**Antigen retrieval**	**Positive control**
LSD1	1B2E5	1:25	mAb #41-3300 (ThermoFisher Scientific)	Nucleus	H1(20 min)	Lung adenocarcinoma
